# Exosome-based therapeutics: emerging extracellular vesicle platforms for targeted delivery in inherited retinal dystrophies and beyond

**DOI:** 10.1097/MS9.0000000000005143

**Published:** 2026-05-14

**Authors:** Sarum A. Khan, Taimoor Ali, Aneeza Hameed, Aiman Tahir, Muhammad A. Qamar

**Affiliations:** a Wah Medical College, Wah Cantt, Pakistan; b Azad Jammu and Kashmir Medical College, Muzaffarabad, Pakistan; c Spinghar Medical University, Kabul, Afghanistan

*To the Editor*,

A recent comprehensive review by Mead and Tomarev on extracellular vesicle (EV) therapy for retinal diseases underscores the unmet translational need for exosome-based platforms in inherited retinal dystrophies (IRDs)[1]. We wish to build on this by examining exosome therapeutics against the limitations of current viral vector approaches. IRDs encompass a diverse array of genetic disorders that primarily impair photoreceptors and the retinal pigment epithelium (RPE), resulting in progressive vision impairment and often irreversible blindness[2]. These conditions exhibit a global prevalence of approximately 1:1380 individuals, impacting an estimated 5.5 million people worldwide, with higher rates in regions of consanguinity and varying socioeconomic contexts that exacerbate access to care[3]. The genetic heterogeneity of IRDs, involving mutations in over 280 genes, combined with the eye’s immune-privileged nature and direct accessibility for interventions like intravitreal or subretinal administration, makes them a relevant candidate for advanced delivery approaches compared to systemic genetic disorders, where delivery barriers and off-target effects are more pronounced[4].

Conventional management of IRDs is predominantly palliative, relying on supportive interventions such as antioxidant vitamins, UV-protective eyewear, low-vision aids, and mobility training, which offer no curative benefit and fail to arrest the inexorable progression of retinal degeneration or restore lost visual function in the majority of affected individuals[5]. Gene augmentation therapies, exemplified by voretigene neparvovec (Luxturna), are confined to a small subset of patients with specific biallelic RPE65 mutations and are hampered by issues including immune responses, short-lived therapeutic expression, intraocular inflammation, and inconsistent inter-patient efficacy, alongside high costs that limit global equity[6]. Adeno-associated viral vectors, the mainstay of current gene delivery, further contend with inherent constraints such as the theoretical potential for insertional mutagenesis, restricted transgene capacity below 4.7 kb (precluding larger genes like ABCA4 or USH2A), suboptimal transduction efficiency in post-mitotic photoreceptors, and scalability hurdles in manufacturing high-titer vectors[7].

Exosome-based therapeutics represent a promising alternative to conventional viral vectors, harnessing naturally secreted EVs as vehicles with low immunogenicity and favorable biocompatibility profiles for targeted payload delivery in retinal pathologies. These lipid-bilayer-enclosed nanoparticles (30–150 nm), originating from endosomal multivesicular bodies, encapsulate a rich cargo of proteins, lipids, messenger RNAs, and microRNAs[8] that orchestrate intercellular signaling through direct membrane fusion or receptor-mediated endocytosis, thereby conferring neuroprotective effects in IRD models such as retinitis pigmentosa (RP) by attenuating apoptotic cascades and dampening inflammatory responses via suppression of pro-inflammatory cytokines like IL-1β, IL-6, TNF-α, and HMGB1[9]. Mesenchymal stem cell-derived exosomes, in particular, exhibit multifaceted mechanisms including miRNA-mediated modulation and oxidative stress mitigation, which collectively preserve photoreceptor viability and retard degeneration in preclinical rodent models of RP and other IRDs[10]. RPE-sourced exosomes additionally counteract oxidative insults and photoreceptor apoptosis by delivering antioxidants and growth factors, while variants from other sources inhibit pathological angiogenesis, thereby curbing macrophage infiltration and neovascular complications that exacerbate retinal damage[10]. In contrast to traditional viral-based modalities, exosomes offer advantages including low immunogenicity, good biocompatibility, and potential for efficient intraocular delivery. Preclinical evidence highlights their efficacy in administration routes like intravitreal injections that achieve retinal penetration with reduced invasiveness compared to subretinal delivery required for many gene therapies.

Exosome-based therapeutics for IRDs remain at the preclinical stage. No exosome therapy has yet entered clinical trials for these conditions, and substantial translational barriers remain. Key unresolved challenges include the standardization of therapeutic cargo loading, long-term storage stability, dosing reproducibility across manufacturing batches, and the regulatory classification of exosome products as biologics or advanced therapy medicinal products. Establishing rigorous pharmacokinetic and safety profiles in large-animal models will be necessary before first-in-human studies can be considered. Coordinated preclinical and regulatory efforts are required before these platforms can be evaluated as viable, cell-free therapeutic options for IRDs and associated ocular vasculopathies.

## Data Availability

All data used are accessible.
